# Modeling and evaluating site and provenance variation in height–diameter relationships for *Betula alnoides* Buch.–Ham. ex D. Don in southern China

**DOI:** 10.3389/fpls.2023.1248278

**Published:** 2023-10-02

**Authors:** Mingyu Yin, Chunsheng Wang, Huan Wang, Qiang Han, Zhigang Zhao, Cheng Tang, Junjie Guo, Jie Zeng

**Affiliations:** ^1^ Research Institute of Tropical Forestry, Chinese Academy of Forestry, Guangzhou, China; ^2^ Agricultural College, Shihezi University, Shihezi, China

**Keywords:** *Betula alnoides*, dummy variable model, genetic improvement, height-diameter relationship, stem form

## Abstract

Tree height (H) and stem diameter at breast height (DBH) (H-D) relationship is correlated with timber yield and quality as well as stability of forest and is crucial in forest management and genetic breeding. It is influenced by not only environmental factors such as site quality and climate factors but also genetic control that is mostly neglected. A dataset of H and DBH of 25 provenances of *Betula alnoides* Buch.–Ham. ex D. Don at four sites was used to model the H-D relationship. The dummy variable nonliner mixed-effect equations were applied to evaluate the effects of sites and provenances on variations of the H-D relationship and to select superior provenances of *B. alnoides*. Weibull equation was selected as the base model for the H-D relationship. The sites affected asymptotes of the H-D curves, and the provenance effect on asymptotes of the H-D curves varied across sites. Taking above-average DBH and lower asymptote of the H-D curves as indicators, five excellent provenances were screened out at each site with a rate of 20%. Their selection gains of individual volume ranged from 1.99% to 29.81%, and their asymptote parameter (*k_j_*) and H-D ratio were 7.17%–486.05% and 3.07–4.72% lower than the relevant total means at four sites, respectively. Genetic selection based on the H-D relationship could promote selection efficiency of excellent germplasms and was beneficial for the large-sized timber production of *B. alnoides*.

## 1 Introduction

The relationship between tree height (H) and stem diameter at breast height (DBH) (H-D) is related to wood volume and quality (Price et al., 2007; Kroon et al., 2008; Richter, 2015), and it also influences the tree vigor and mechanical stability (Robichaud and Methven, 1991; Peltola et al., 2000; Valinger and Fridman, 2011). The H-D relationship is of important applications in forest measurement, volume or biomass estimation, and forest management (Gould and Marshall, 2010; Ducey, 2012; Temesgen et al., 2014; Yih et al., 2017). All sorts of the H-D equations have been developed up to now, such as power (Stage, 1975), hyperbolic (Scaranello et al., 2012), exponential (Wykoff et al., 1982), monomolecular (Pearl and Reed, 1920), logistic (Huang and Titus, 1992), and Weibull (Yang et al., 1978) models, which lay a foundation for the understanding of the H-D relationship.

It is said that introduction of biotic and abiotic factors into H-D basic equations can improve their prediction accuracy (Zhang et al., 2019) and promote the cognition on driving forces for the variance of the H-D relationship. Many studies have been documented on modeling the H-D relationship, in which stand characteristics, site quality, and geographical and climate factors are usually taken into consideration (Wang et al., 2006; Feldpausch et al., 2011; Hulshof et al., 2015; Trouvé et al., 2015; Vizcaíno-Palomar et al., 2016). Homeier et al. (2010) found out that H of forest stands decreased more than DBH with increasing altitude in the European forests. It was indicated from the study of Bošeľa et al. (2013) that good soil nutrient and climatic conditions also sped up the growth of height and DBH and thus influenced the H-D relationship of Norway spruce [*Picea abies* (L.) Karst.]. Zhang et al. (2019) verified that higher stand density always promoted growth of H more than that of DBH and built more slender stem of Chinese fir [*Cunninghamia lanceolata* (Lamb.) Hook.]. Although genetic influences on the H-D relationship have been given less attention to, only a few studies have been reported, in which the significant effects of genetic entry on the H-D relationship are shown. Buford (1986) found out that the asymptotes of the H-D curves were different among the provenances of loblolly pine (*Pinus taeda* L.). The study of Sabatia and Burkhart (2013) on loblolly pine demonstrated that the asymptotes of the H-D curves increased with increasing levels of genetic improvement. These studies all demonstrate the importance of incorporating genetic factors into models about the H-D relationship.

As to the accurate evaluation of the genetic effect on the H-D relationship, there are still several challenges. First, the genetic effect on the H-D relationship usually varies with tree species (Vizcaíno-Palomar et al., 2016), although only a small number of studies have been reported, and for a few coniferous species (Buford and Burkhart, 1987; Weng et al., 2008; Egbäck et al., 2018). Second, large-sized samples under well-designed experiments are also essential to model and illustrate the genetic effect on the H-D relationship. Third, the interaction between genotype and site normally exists in the practice, and incorporating site effect into the H-D model is thus also necessary.

Up to now, few case studies of germplasm selection have been reported taking H-D relationship into consideration. In the previous studies, H and DBH are usually used as core and independent traits for the selection of elite germplasm (Vergara et al., 2004). This causes disproportional improvement for both traits and further reduces the breeding efficiency (e.g., Carson et al., 1999; Andersson et al., 2007; Sabatia and Burkhart, 2013). Introducing the H-D relationship into the selection process may avoid this disadvantage and promote selection efficiency of excellent germplasms.

*Betula alnoides* Buch.–Ham. ex D. Don is a fast-growing valuable tree species in the family Betulaceae and is indigenous to the warm subtropical and tropical regions in Southeast Asia and southern China (Zeng et al., 2003). The typical rotation age is 20 years for this species, and its wood is well known for its beautiful texture, moderate density, and excellent manufacturing characteristics and is extensively used for high-grade floor, furniture, and overlaid veneer making (Wang et al., 2016). Its bark is rich in secondary metabolites with anti-inflammatory and lipid-lowering functions and is also an ideal ingredient for traditional medicine (Sur et al., 2002; Raj et al., 2015). Strong adaptability to different environments has resulted in the distribution of the species across wide ranges of soil types, altitudes, and climate conditions (Zeng et al., 1999). To meet the increasing demand for its high-quality timber, *B. alnoides* has been widely planted, and more than 220,000 ha of its plantations have been established in Yunnan, Guangxi, Guangdong, and Fujian Provinces of China up to now. To select excellent germplasms for plantation forestry, many provenances and family trials have been established to reveal the genetic variation of growth and stem quality traits at multiple sites. Guo et al. (2008) and Yang et al. (2012) used H and DBH to select excellent provenances for *B. alnoides* at single site. Yin et al. (2019) conducted multi-site joint selection on basis of H, DBH, individual volume, and stem quality. Although some excellent provenances of *B. alnoides* are screened out, neglection of the H-D relationship in these studies may result in disproportional improvement for H and DBH and thus decrease its breeding efficiency. On the basis of the provenance and family selection trials of *B. alnoides* at Mengla, Pingxiang, Hua’an, and Changning sites in southern China, the objectives of the present study are (1) to simulate the H-D curves with considering the effects of provenance and site using dummy variable approach and (2) to select superior provenances taking the H-D relationship into consideration.

## 2 Materials and methods

### 2.1 Materials

**Figure 1:**
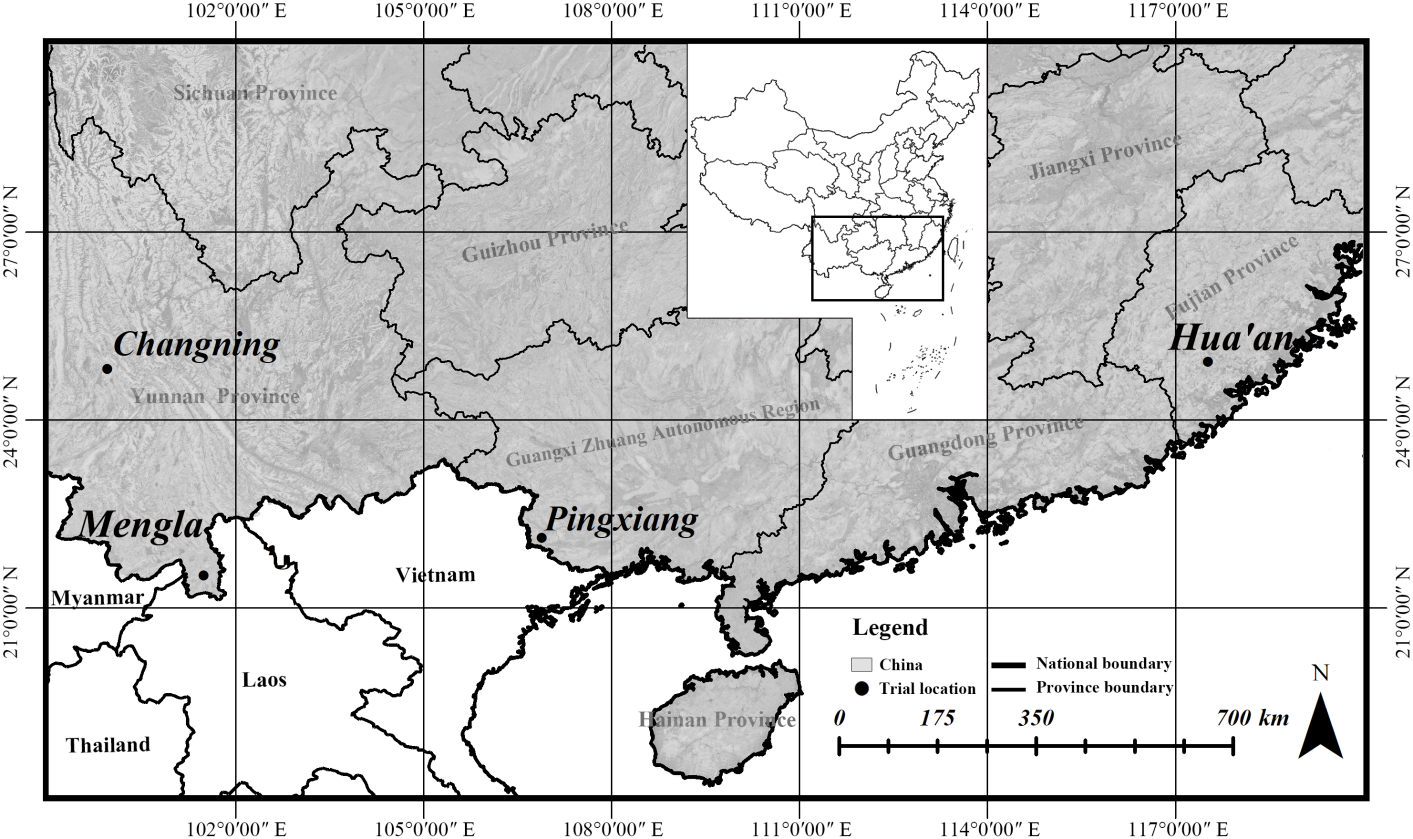
Locality of provenance family trials for *Betula alnoides*.

The provenance family trials of *B. alnoides* were established at Mengla and Pingxiang in 2002, Hua’an in 2003, and Changning in 2007 (**Figure 1**), where 400, 386, 280, and 250 half-sib families were involved, respectively, all from 25 provenances. Their seeds were collected from the natural forests in Yunnan and Guangxi, China, and their seedlings were raised separately at a nursery near each site. Specimens of each family were made when sampling in the natural forests, and species identification was done by Dr. Jie Zeng. Voucher specimens of 25 provenances were deposited at the Herbarium of the Research Institute of Tropical Forestry, Chinese Academy of Forestry, Guangzhou, China, and their information were shown in **Supplementary Table 1**. The information of geographic locations and climatic conditions at the four sites and geographic locations of 25 provenances could be seen detailedly in our previous study (Yin et al., 2019). The seedlings were planted in randomized complete block design with single individual plots and 12 to 19 blocks at a spacing of 2 m × 3 m.

### 2.2 Measurement of tree growth traits

**Figure 2:**
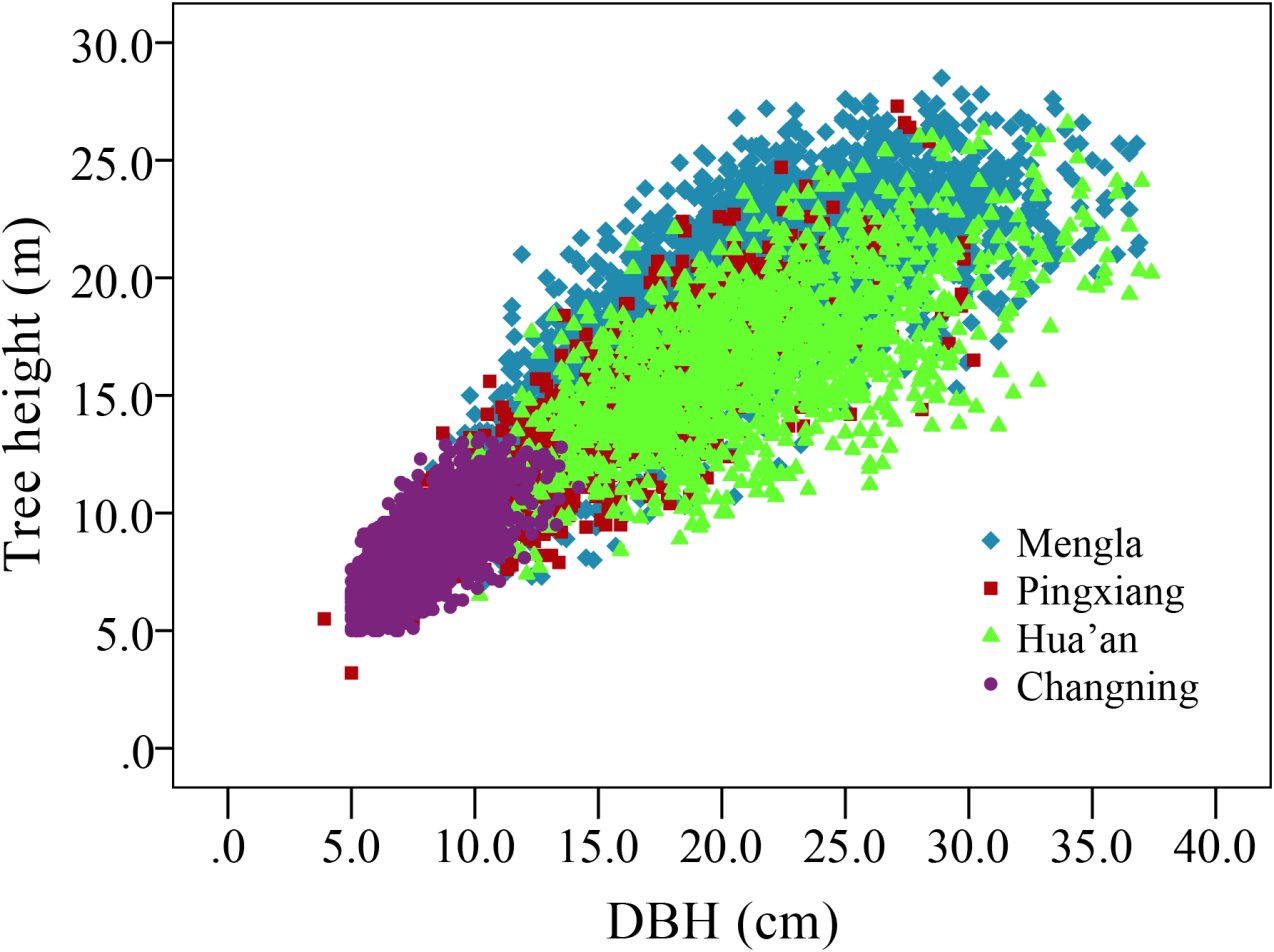
Scatter plot of tree height and stem diameter at breast height (DBH) of *Betula alnoides* at four sites.

Tree growth was investigated at four sites in late 2017 to early 2018. The blocks with survival rates lower than 60% were discarded at each site so as to keep consistency of survival rate among provenances and sites and thus avoid the impact of mortality on the H-D relationship. The current survival rate ranges from about 63.16% to 66.61% at four sites. All trees in well-reserved blocks were measured for DBH to the nearest 0.1 cm using a diameter tape and for H to the nearest 0.1 m using a Vertex IV Altimeter (Haglöf Sweden AB, Västernorrland, Sverige). A total of 7,724 trees were used for model analysis, including 3,403, 953, 1,438, and 1,930 trees at the ages of 15, 15, 14, and 10 years in Mengla, Pingxiang, Hua’an, and Changning sites, respectively. Variance analyses (ANOVA) and Tukey’s multiple range tests were conducted to estimate the differences in DBH and height among sites and provenances. The scatter plots of H and DBH showed a curvilinear relationship at all four sites (**Figure 2**).

### 2.3 Selection and extension of statistical models

Ten commonly used H-D equations (**Table 1**), including two linear and eight nonlinear ones, were used to model the H-D relationships. For evaluating the model performances, the goodness of fit for each model was then assessed with mean absolute error (MAE), Akaike’s information criterion (AIC), adjusted coefficient of determination (R^2^), largest log-likelihood (LL) and paired *t*-test (Liu et al., 2017). The equation with a high goodness of fit was selected as the base model for further analysis.

**Table 1:** Parameter estimates for the candidate models.

Models		Reference	Model parameters		
Codes	Expression		*a*	*b*	*c*
(1)	H=a+bDBH	(Lei et al., 2009)	3.503 (0.076)*	0.692 (0.004)*	
(2)	$H^{-1}=a+bDBH^{-1}$	(Vanclay, 1995)	0.021 (0.000)*	0.742 (0.004)*	
(3)	$H=a+bDBH+cDBH^{2}$	(Henriksen, 1950)	−0.135 (0.131)*	1.246(0.017)*	−0.017 (0.001)*
(4)	$H=1.3+a{\left(1-e^{-bDBH}\right)}^{c}$	(Richards, 1959)	25.357 (0.043)*	0.070 (0.003)*	1.459 (0.038)*
(5)	$H=1.3+aDBH^{b}$	(Stage, 1975)	1.539 (0.027)*	0.786 (0.006)*	
(6)	$H=1.3+e^{\left(a+\frac{b}{DBH+1}\right)}$	(Zhang et al., 2014)	3.448 (0.006)*	−13.081 (0.105)*	
(7)	$H=1.3+\frac{aDBH}{b+DBH}$	(Li et al., 1994)	61.280 (1.489)*	54.634 (1.818)*	
(8)	$H=1.3+a(1-e^{-bDBH^{c}})$	(Yang et al., 1978)	23.871 (0.360)*	0.024 (0.001)*	1.311 (0.020)*
(9)	$H=a{\left(1-e^{-bDBH}\right)}^{c}$	(Richards, 1959)	27.832 (0.510)*	0.059 (0.003)*	1.187 (0.030)*
(10)	$H=a(1-e^{-bDBH^{c}})$	(Yang et al., 1978)	26.737 (0.504)*	0.037 (0.001)*	1.144 (0.018)*

Values in brackets are standard error; H, tree height; DBH, stem diameter at breast height; and * indicates that the parameter was significantly different from zero at 0.05 level.

Site and provenance variables usually affected the asymptotes rather than the slopes of the H-D equations (Buford, 1986; Fu et al., 2016; Zhang et al., 2019); they were thus introduced step by step into the asymptote parameter of the base model in the present study. Here, the extended models for Equations (1) and (8) in **Table 1** were taken as examples for linear and nonlinear equations, respectively:


(11) $$\text{H}=\left(\varphi_{0}+\sum_{i=1}^{3}x_{i}S_{i}+\sum_{i=1}^{24}k_{j}P_{j}\right)+\varphi_{1}\text{DBH}+\epsilon$$

(12) $$\text{H}=1.3+\left(\varphi_{0}+\sum_{i=1}^{3}x_{i}S_{i}+\sum_{i=1}^{24}k_{j}P_{j}\right)\left(1-e^{-\varphi_{2}{\text{DBH}}^{\varphi_{1}}}\right)+\epsilon$$

where *H* and *DBH* are the tree height and stem diameter at breast height, respectively; $\varphi_{0}$ , $\varphi_{1}$ , and $\varphi_{2}$ , are basic parameters; $x_{i}$ and $k_{j}$ are dummy parameters; and ϵ is error term. $S_{i}$ denotes the site dummy variable with *i* = 1, 2, and 3. Changning site is taken as a reference, and Mengla, Pingxiang, and Hua’an are set as dummy variables: $S_{1}$ = 1 denotes Mengla site and 0 for the rest of sites; $S_{2}$ = 1 denotes Pingxiang site and 0 for the rest of sites; $S_{3}$ = 1 denotes Hua’an site and 0 for the rest of sites; and the Changning site is represented by $S_{1}$ = $S_{2}$ = $S_{3}$ = 0. $P_{j}$ denotes the provenance dummy variable with *j* = 1, 2, 3, …, 24. Provenance Y is taken as a reference, and other provenances are set as dummy variables: $P_{1}$ = 1 denotes provenance A and 0 for the rest of provenances; $P_{2}$ =1 denotes provenance B and 0 for the rest of provenances; and so on…. $P_{24}$ = 1 denotes provenance X and 0 for the rest of provenances; and the provenance Y is represented by $P_{1}$ = $P_{2}$ = ⋯ = $P_{24}$ = 0. The likelihood-ratio test (LRT) (Fang and Bailey, 2001) was used to estimate whether the site or provenance variables influenced the equation parameters significantly:


(13) $${\text{LRT}}_{i-j}=2({\text{LL}}_{i}-{\text{LL}}_{j})$$

where ${\text{LL}}_{i}$ and ${\text{LL}}_{j}$ are largest log-likelihood of extended model *i* and base model *j*. Compared with the critical values for chi-squared distribution, if $X_{i-j}^{2}>X_{\left(n_{i}-n_{j},0.05\right)}^{2}$ , there exist significant differences between model *i* and model *j* at 0.05 level. The model parameters then are influenced significantly by site or provenance variables, where $n_{i}$ and $n_{j}$ are the degrees of freedom of extended model *i* and base model *j*, respectively.

Block was then introduced into the dummy model as random effects to analyze the H-D allometry. A method of Davisian and Giltinan (1995) was used to account for the within-tree heteroscedasticity and autocorrelation in the variance–covariance matrix ( ${\text{R}}_{ijlm}$ ) of the error term ( $\epsilon_{ijlm}$ ):


(14) $${\text{R}}_{ijlm}=\sigma^{2}{\text{G}}_{ijlm}^{0.5}{Г}_{ijlm}{\text{G}}_{ijlm}^{0.5}$$

where ${\text{R}}_{ijlm}$ is a variance–covariance matrix of the error term ( $\epsilon_{ijlm}$ ) in the *m*th tree nested within *l*th block for the *j*th provenance at the *i*th site; $\sigma^{2}$ is a scaling factor for error dispersion, given by a value of residual variance of the estimated model; and ${\text{R}}_{ijlm}$ and ${Г}_{ijlm}$ are $n_{ijlm}$ × $n_{ijlm}$ diagonal matrix explaining the variance of within-tree heteroscedasticity and autocorrelation structure of errors. Power variance fuction (Fu et al., 2016) was used to reduce heterogeneity in variance:


(15) $${\text{var}}_{\left(\epsilon \right)}=\sigma^{2}x^{2\gamma }+\epsilon^{\prime }$$

where *x* is fitted values as the selected predictor, γ is the parameter to be estimated, and $\epsilon^{\prime }$ is an error term. The all parameters in the nonlinear mixed-effects (NLME) models were estimated with restricted maximum likelihood implemented in R software “nlme” package (Pinheiro et al., 2017).

On the basis of NLME models, the curves of the H-D relationship at four sites and of 25 provenances at each site were simulated to assess variation among sites and provenances. Considering the visualization and simplicity for the H-D relationship in line graphs as well as subsequent provenance selection, the 25 provenances were clustered into several groups through the system clustering method based on Euclidean distance of asymptote parameter $k_{j}$ at each site. The H-D relationship of groups was then showed in a line graphs and used for comparison and selection for provenances.

### 2.4 Excellent provenances selection

The H-D relationship was further involved in the selection of excellent germplasm. Because the large-sized timber production is the target of management for valuable tree species and lower asymptote of the H-D relationship at a given DBH is important for wood quality and tree stability, above-average DBH growth and lower asymptote of the H-D curves were thus used as the indicators. The provenances with above-average DBH were ranked by their asymptote parameter in the H-D equations, and excellent provenances with lower values of asymptote parameter were then selected with a rate of 20% at each site, i.e., five provenances of the total 25 were selected. The individual volumes were used to evaluate the effect of selection and were calculated as volume = 0.45/4πDBH^2^ × H (Wang et al., 2013), and selection gains were calculated as selection gain = (selected mean − total mean)/total mean × 100%.

All data analyses and modeling were performed using R software, SPSS 13.0, and Microsoft Excel 2010.

## 3 Results

### 3.1 Site and provenance variations of DBH and tree height

There were significant differences in DBH and H of *B. alnoides* among the four sites at 0.05 level (**Table 2**). The DBH and height performed better at Mengla and Hua’an than at the other two sites. At each site, both traits differed significantly among provenances, and well-performed provenances varied with sites, which demonstrated obvious interaction between sites and provenances. Provenance A, G, and B performed the best in both growth traits at Mengla, Hua’an, and Changning, respectively; and provenance V and R performed the best in H and DBH at Pingxiang, respectively.

**Table 2:** Mean values of stem diameter at breast height (DBH) and tree height of 25 *Betula alnoides* provenances at four sites.

Provenances	Mengla			Pingxiang			Hua’an			Changning		
	*N*	DBH (cm)	Height (m)	*N*	DBH (cm)	Height (m)	*N*	DBH (cm)	Height (m)	*N*	DBH (cm)	Height (m)
A	114	22.91 (1.11)a	20.61 (0.74)a	42	11.65 (0.81)f	11.93 (0.8)bcde	67	19.56 (1.77)ab	16.34 (1.37)ab	108	7.59 (0.13)ab	8.46 (0.14)abc
B	129	21.53 (0.92)abcd	18.82 (0.58)abcdef	36	12.85 (0.85)cdef	12.05 (0.62)bcde	43	19.16 (0.84)ab	16.12 (0.58)ab	78	8.72 (0.40)a	8.95 (0.36)a
C	149	20.99 (0.45)abcd	19.77 (0.31)abcdef	35	12.33 (0.57)def	11.73 (0.51)cde	53	19.34 (0.68)ab	16.13 (0.41)ab	67	7.88 (0.23)ab	8.25 (0.21)abc
D	111	21.63 (0.42)abcd	20.25 (0.29)ab	34	12.94 (0.71)bcdef	12.57 (0.53)abcde	65	18.92 (0.83)ab	16.16 (0.49)ab	84	7.61 (0.15)ab	8.28 (0.15)abc
E	135	20.45 (0.42)abcd	18.45 (0.29)bcdef	44	16.89 (0.6)abc	14.88 (0.64)abc	71	20.94 (0.49)ab	16.29 (0.39)ab	102	7.79 (0.14)ab	8.24 (0.16)abc
F	131	21.2 (0.49)abcd	20.01 (0.32)abcd	32	11.88 (0.73)ef	11.24 (0.67)e	54	20.71 (1.32)ab	15.96 (0.43)ab	59	7.74 (0.26)ab	8.08 (0.20)abc
G	133	18.98 (0.44)cd	18.52 (0.32)bcdef	33	13.12 (0.96)bcdef	13.18 (0.91)abcde	61	23.46 (1.30)a	19.02 (0.88)a	–	–	–
H	149	21.13 (0.39)abcd	19.33 (0.27)abcdef	36	13.81 (1.08)abcdef	12.48 (0.77)abcde	49	20.13 (0.69)ab	16.71 (0.37)ab	78	7.99 (0.26)ab	8.66 (0.29)abc
I	133	21.77 (0.52)ab	20.08 (0.34)abc	39	14.05 (0.95)abcdef	13.42 (0.83)abcde	64	20.66 (0.86)ab	16.44 (0.52)ab	61	7.16 (0.44)b	8.17 (0.55)abc
J	146	20.72 (0.42)abcd	18.95 (0.29)abcdef	43	12.17 (0.65)ef	12.17 (0.61)abcde	54	21.58 (0.55)ab	16.83 (0.39)ab	58	8.26 (0.26)ab	8.91 (0.22)a
K	134	21.45 (0.38)abcd	19.44 (0.26)abcdef	41	15.12 (0.56)abcdef	13.77 (0.50)abcde	51	20.08 (0.59)ab	16.54 (0.45)ab	76	7.87 (0.50)ab	8.79 (0.51)abc
L	148	21.7 (0.43)abc	19.98 (0.28)abcde	32	12.26 (0.65)ef	11.33 (0.49)de	55	17.94 (0.96)b	15.88 (0.69)b	92	7.84 (0.12)ab	8.39 (0.12)abc
M	138	20.4 (0.43)abcd	19.52 (0.29)abcdef	49	13.32 (0.85)bcdef	12.93 (0.76)abcde	41	21.01 (0.71)ab	16.85 (0.47)ab	104	7.68 (0.15)ab	8.27 (0.15)abc
N	151	20.67 (0.43)abcd	18.64 (0.31)abcdef	32	12.12 (0.69)ef	11.88 (0.74)bcde	64	20.90 (1.43)ab	15.68 (0.82)b	82	7.90 (0.17)ab	8.38 (0.16)abc
O	114	20.34 (0.51)abcd	18.01 (0.42)ef	39	16.59 (0.83)abcd	13.75 (0.49)abcde	46	20.42 (0.61)ab	17.05 (0.53)ab	98	7.74 (0.15)ab	8.04 (0.14)abc
P	130	20.79 (0.37)abcd	18.74 (0.27)abcdef	51	17.06 (0.59)abc	14.62 (0.44)abcde	59	22.80 (0.78)a	18.24 (0.50)ab	61	7.91 (0.27)ab	8.15 (0.23)abc
Q	128	19.85 (0.86)bcd	18.39 (0.49)bcdef	30	15.26 (0.79)abcdef	13.39 (0.52)abcde	64	23.26 (0.78)a	17.14 (0.48)ab	76	7.92 (0.49)ab	8.84 (0.55)ab
R	155	19.89 (0.35)bcd	18.53 (0.26)bcdef	48	17.99 (0.67)a	14.62 (0.45)abcde	76	22.38 (0.47)ab	16.97 (0.3)ab	68	7.79 (0.17)ab	8.29 (0.15)abc
S	136	19.88 (0.83)bcd	18.04 (0.73)def	29	16.02 (1)abcde	13.96 (0.69)abcde	73	19.60 (1.02)ab	15.15 (0.54)b	76	7.41 (0.30)ab	8.09 (0.42)abc
T	120	20.33 (0.64)abcd	18.27 (0.45)bcdef	24	15.92 (0.78)abcdef	14.47 (0.61)abcde	52	20.68 (0.77)ab	15.90 (0.48)b	76	8.17 (0.31)ab	8.48 (0.30)abc
U	151	18.88 (0.34)d	18 (0.29)f	39	17.01 (0.47)abc	14.24 (0.41)abcde	61	22.31 (0.64)ab	17.02 (0.46)ab	80	7.34 (0.17)ab	7.92 (0.19)bc
V	140	19.58 (0.34)bcd	18.18 (0.27)cdef	47	17.22 (0.51)ab	15.58 (0.44)a	51	21.05 (0.75)ab	15.16 (0.51)b	84	8.23 (0.24)ab	8.53 (0.19)abc
W	140	20.79 (0.42)abcd	18.53 (0.3)bcdef	39	16.72 (0.58)abc	14.79 (0.55)abcd	64	22.24 (0.40)ab	16.28 (0.26)ab	63	8.00 (0.23)ab	8.18 (0.17)abc
X	159	19.28 (0.33)bcd	18.39 (0.28)bcdef	46	15.85 (0.54)abcdef	14.14 (0.46)abcde	45	20.40 (0.47)ab	15.81 (0.29)b	101	7.30 (0.10)ab	7.90 (0.10)c
Y	129	20.64 (0.38)abcd	18.97 (0.27)abcdef	33	17.2 (0.61)ab	15.36 (0.48)ab	55	21.23 (0.41)ab	16.44 (0.26)ab	98	7.61 (0.14)ab	7.94 (0.13)bc
Means	136	20.58 (0.09)B	19.00 (0.06)A	38	15.57 (0.15)C	13.89 (0.12)C	58	21.12 (0.14)A	16.46 (0.09)B	80	7.74 (0.04)D	8.26 (0.04)D

Values in brackets are standard error; N, number of trees per provenance; A, Mengla; B, Yuanyang; C, Mojiang; D, Jinghong; E, Xichou; F, Zhenyuan; G, Tengchong; H, Jinggu; I, Ruili; J, Fengqing; K, Pingbian; L, Jiangcheng; M, Shuangjiang; N, Lancang; O, Lingyun; P, Longzhou; Q, Donglan; R, Tianlin; S, Debao; T, Tiane; U, Pingguo; V, Baise; W, Tianyang; X, Jingxi; Y, Napo; –, missing value. Means with standard error in parenthesis were of signiﬁcant difference at 0.05 level according to Tukey’s multiple comparison tests if followed by wholly different capital and small letters in the same row and column, separately.

### 3.2 Nonlinear mixed-effect H-D model

All parameters estimated in each H-D candidate function were of significant difference from zero at 0.05 level (**Table 1**). The paired *t*-test showed that there was no significant difference between observed values and predicted values of each model except model (10). The MAE and AIC of the Weibull model [Equation (8)] were the lowest, and its coefficient of determination (R^2^) was the greatest among all equations (**Table 3**). Weibull model also satisfied the biological assumption that H is 1.3 m (breast height) when DBH equals to zero for the H-D relationship (Bi et al., 2012), it was thus selected as the base model.

**Table 3:** Paired *t*-test of predicted and observed values and goodness-of-fit statistics for the candidate models.

Models	Paired differences				Goodness of fit		
	Mean	SE	*t*-value	Significance level	MAE	AIC	R^2^
(1)	0.036	0.056	0.651	0.515	1.935	11,529.926	0.808
(2)	−0.001	0.00035	−1.848	0.065	1.851	11,041.354	0.833
(3)	−0.057	0.052	−1.094	0.274	1.791	10,536.668	0.836
(4)	−0.023	0.052	−0.445	0.657	1.789	10,545.103	0.836
(5)	0.066	0.054	1.225	0.221	1.881	11,121.999	0.820
(6)	−0.018	0.053	−0.344	0.731	1.857	10,760.911	0.830
(7)	0.098	0.053	1.866	0.062	1.831	10,805.137	0.829
(8)	0.000	0.052	−0.008	0.994	1.784	10,528.054	0.837
(9)	−0.020	0.052	−0.389	0.697	1.798	10,580.029	0.835
(10)	0.107	0.052	2.065	0.039*	1.797	10,571.010	0.836

SE, standard error; MAE, mean absolute error; AIC, Akaike’s information criterion; R^2^, coefficient of determination; and * indicates that the differece between predicted and observed values was significant at 0.05 level. The predictive value of response variables H^−1^ was converted to H of model (2), and, then, the goodness-of-fit parameters were calculated.

Site and provenance variable was introduced as dummy variables step by step into the asymptote parameter of the base model [Equation (8)], and the random parameters of block were then added to the asymptote and slope of DBH to develop site-level NLME model [Equation (16)] as follows:


(16) $$H_{ilm}=1.3+\left(\varphi_{0}+\sum_{i=1}^{3}x_{i}S_{i}+\mu_{0l}\right)\left(1-e^{-\varphi_{2}DBH^{\left(\varphi_{1}+\mu_{1l}\right)}}\right)+\epsilon_{ilm}$$

Considering the interaction between sites and provenances, provenance dummy variables were introduced into the base model [Equation (8)] at each site, the provenance-level NLME model was then shown as follows:


(17) $$H_{jlm}=1.3+\left(\varphi_{0}+\sum_{i=1}^{24}k_{j}P_{j}+\mu_{0l}\right)\left(1-e^{-\varphi_{2}DBH^{\left(\varphi_{1}+\mu_{1l}\right)}}\right)+\epsilon_{jlm}$$

where $\mu_{0l}$ and $\mu_{1l}$ are the random effects on asymptote and slope caused by the *l*th block, and $\mu_{0l}$ ~*N*(0, $\sigma_{0block}^{2}$ ), $\mu_{1l}$ ~*N*(0, $\sigma_{1block}^{2}$ ).

**Figure 3:**
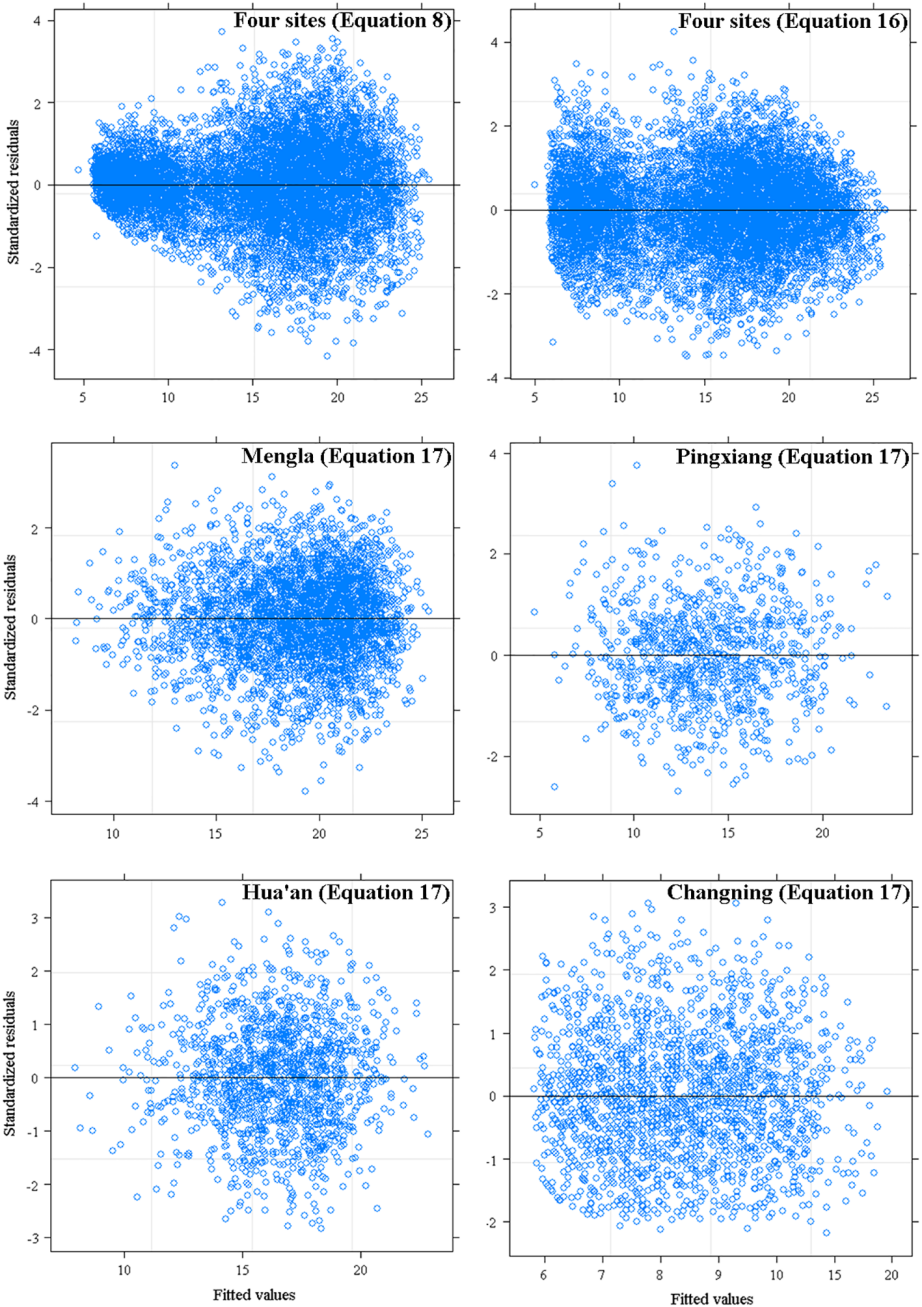
Residuals plots of base model [Equation (8)], site-level [Equation (16)] and provenance-level mixed-effects model [Equation (17)] against fitted values of tree height.

The simulation results and the model performance of the extended models are shown in **Tables 4**, **5**, respectively. The performance of site-level NLME model [Equation (16)] and provenance-level NLME model [Equation (17)] were significantly improved with a smaller MAE and AIC values and a larger R^2^ and LL values than the base model [Equation (8)]. The LRT test results also demonstrated that asymptote parameters of the models were influenced significantly by site variables and provenance variables at four sites (*P<* 0.05). The residual scatter diagram of Equation (8) inferred obvious increase trend of predicted H with increasing DBH. Because the heteroscedasticity was effectively accounted by the power variance function [Equation (15)], this trend disappeared with NLME models [Equations (16) and (17) at four sites] (**Figure 3**).

**Table 4:** Parameter estimates of the nonlinear mixed-effects model at site-level [Equation (16)] and provenance-level [Equation (17)] for *Betula alnoids*.

	Parameter estimates	Parameter definition	Site model [Equation (16)]	Provenance model at Mengla [Equation (17)]	Provenance model at Pingxiang [Equation (17)]	Provenance model at Hua’an [Equation (17)]	Provenance model at Changning [Equation (17)]
Fixed-effects parameters	*x*_1_	Mengla	4.388 (0.278)***				
	*x*_2_	Pingxiang	0.493 (0.228)*				
	*x*_3_	Hua’an	−0.002 (0.238)				
	*k*_1_	A		1.041 (0.515)*	2.374 (1.548)	0.753 (1.679)	0.853 (0.222)***
	*k*_2_	B		−0.577 (0.501)	−0.212 (1.040)	1.009 (0.841)	0.312 (0.420)
	*k*_3_	C		0.910 (0.317)**	−0.047 (0.953)	0.786 (0.695)	0.262 (0.264)
	*k*_4_	D		1.087 (0.303)**	0.530 (1.090)	1.178 (0.958)	0.465 (0.223)*
	*k*_5_	E		−0.607 (0.329)	−0.750 (0.809)	−0.286 (0.569)	0.062 (0.228)
	*k*_6_	F		1.040 (0.327)**	−1.335 (1.142)	−0.568 (1.156)	−0.061 (0.347)
	*k*_7_	G		0.722 (0.341)*	1.268 (1.422)	2.632 (1.377)	−
	*k*_8_	H		0.167 (0.302)	−0.426 (1.023)	1.284 (0.725)	0.460 (0.320)
	*k*_9_	I		1.128 (0.313)***	0.373 (1.162)	0.318 (0.800)	0.735 (0.555)
	*k*_10_	J		−0.068 (0.320)	1.749 (1.170)	0.059 (0.662)	0.654 (0.279)*
	*k*_11_	K		0.136 (0.295)	−0.332 (0.795)	0.611 (0.699)	0.865 (0.573)
	*k*_12_	L		0.616 (0.314)*	−1.072 (0.980)	1.600 (0.985)	0.329 (0.216)
	*k*_13_	M		1.008 (0.315)**	1.175 (1.193)	0.612 (0.747)	0.363 (0.222)
	*k*_14_	N		−0.058 (0.304)	0.381 (1.144)	−0.905 (0.920)	0.312 (0.225)
	*k*_15_	O		−1.068 (0.383)**	−1.998 (0.932)*	1.413 (0.749)	−0.018 (0.225)
	*k*_16_	P		−0.303 (0.296)	−1.026 (0.706)	1.721 (0.677)*	−0.004 (0.275)
	*k*_17_	Q		−0.615 (0.596)	−1.880 (1.078)	−0.546 (0.889)	0.911 (0.466)*
	*k*_18_	R		−0.181 (0.309)	−2.043 (0.782)**	0.089 (0.471)	0.317 (0.224)
	*k*_19_	S		−0.587 (0.537)	−0.795 (1.083)	−0.976 (0.812)	0.475 (0.460)
	*k*_20_	T		−0.753 (0.432)	0.318 (1.011)	−0.467 (0.668)	0.299 (0.323)
	*k*_21_	U		−0.023 (0.317)	−2.152 (0.730)**	0.007 (0.627)	0.342 (0.253)
	*k*_22_	V		−0.371 (0.304)	0.514 (0.691)	−2.210 (0.752)**	0.171 (0.246)
	*k*_23_	W		−0.682 (0.324)*	−0.459 (0.749)	−0.953 (0.476)*	−0.111 (0.265)
	*k*_24_	X		0.226 (0.307)	−0.329 (0.734)	−0.404 (0.486)	0.187 (0.207)
	*φ*_0_	Asymptote	23.760 (0.542)***	22.404 (0.375)***	29.345 (3.866)***	25.506 (3.157)***	11.636 (0.805)***
	*φ*_1_	Slope	1.101 (0.020)***	1.556 (0.050)***	1.158 (0.068)***	0.971 (0.097)***	1.411 (0.087)***
	*φ*_2_	Rate	0.037 (0.001)***	0.015 (0.002)***	0.024 (0.002)***	0.048 (0.006)***	0.051 (0.004)***
Variance components	*σ*_0block_		0.000	0.609	1.494	0.741	0.000
	*σ*_1block_		0.010	0.017	0.000	0.000	0.000
	*σ*		0.186	3.128	0.308	0.362	0.159
	*γ*		0.885	−0.106	0.670	0.681	0.857

A-Y, the number of provenances; A, Mengla; B, Yuanyang; C, Mojiang; D, Jinghong; E, Xichou; F, Zhenyuan; G, Tengchong; H, Jinggu; I, Ruili; J, Fengqing; K, Pingbian; L, Jiangcheng; M, Shuangjiang; N, Lancang; O, Lingyun; P, Longzhou; Q, Donglan; R, Tianlin; S, Debao; T, Tiane; U, Pingguo; V, Baise; W, Tianyang; X, Jingxi; Y, Napo; −, missing data; *, **, and *** indicates that the difference was significant at 0.05, 0.01, and 0.001 levels, respectively.

**Table 5:** Comparison of goodness-of-fit statistics for base model [Equation (8)] and nonlinear mixed-effects (NLME) model [Equations (16) and (17)] at site-level and provenance-level.

Models	Level	MAE	R^2^	AIC	LL	LRT
Base model [Equation (8)]	Site model	1.784	0.837	10528.054	−5261.027	
	Provenance model at Mengla	1.859	0.608	5,860.973	−2,927.487	
	Provenance model at Pingxiang	1.559	0.713	1,338.962	−666.481	
	Provenance model at Hua’an	1.996	0.440	2,679.407	−1,336.704	
	Provenance model at Changning	0.802	0.639	−5.195	5.598	
NLME model [Equation (16)]	Site model	1.596	0.852	9,247.579	−4,620.790	LRT _8–16 = _2404.19*
NLME model [Equation (17)]	Provenance model at Mengla	1.796	0.633	5,629.552	−2,811.776	LRT _8–17 = _231.422*
	Provenance model at Pingxiang	1.417	0.770	1,127.027	−560.513	LRT _8–17 = _211.935*
	Provenance model at Hua’an	1.918	0.481	2,565.721	−1279.860	LRT _8–17 = _113.686*
	Provenance model at Changning	0.787	0.648	−59.411	32.705	LRT _8–17 = _54.216*

MAE, mean absolute error; R^2^, coefficient of determination; AIC, Akaike’s information criterion; LL, largest log-likelihood; LRT, likelihood-ratio test; * indicates that the difference was significant at 0.05 level.

### 3.3 Site and provenance variations of the H-D relationship

**Figure 4:**
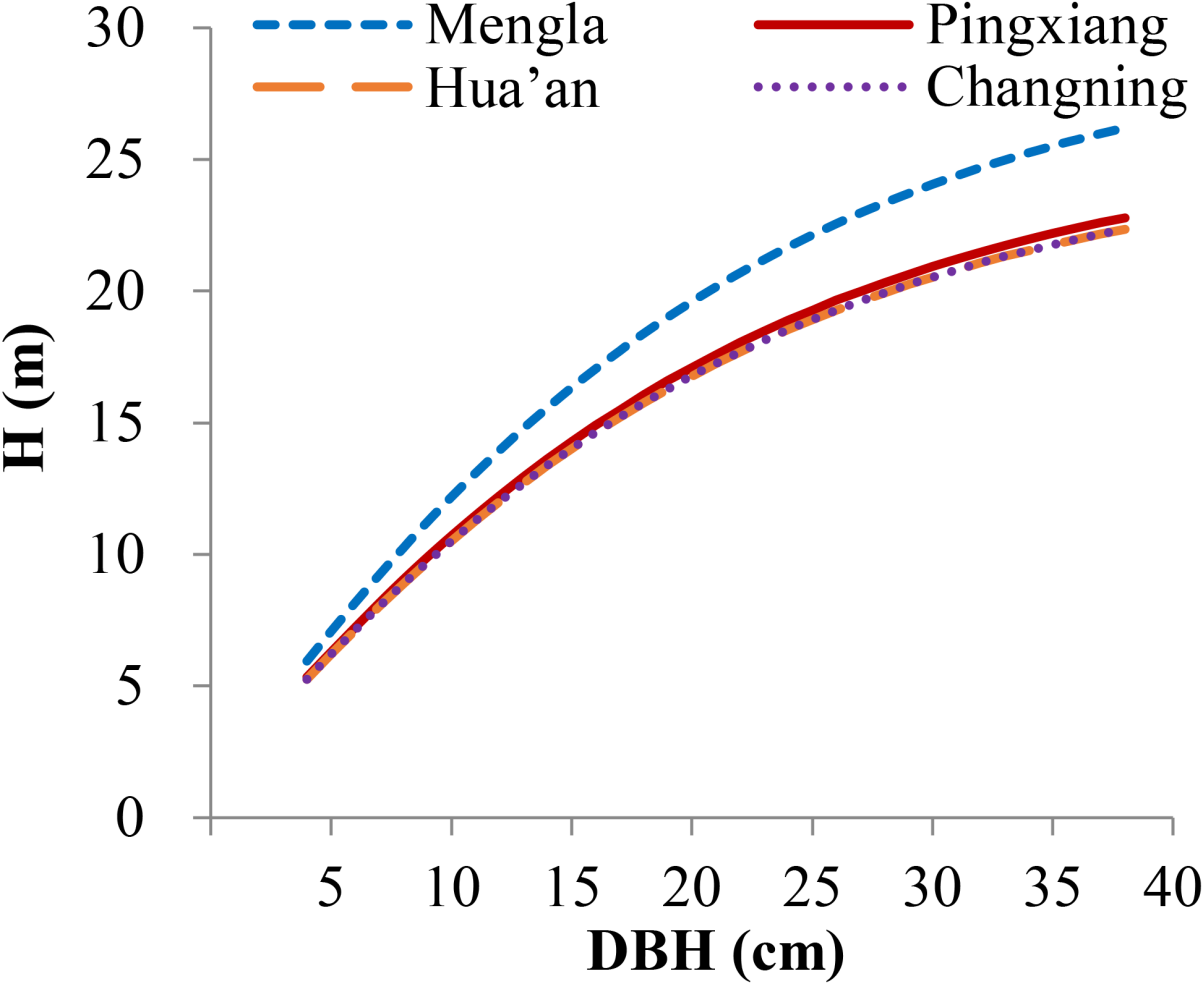
Simulation plots of height–diameter curves for *Betula alnoides* based on site-level nonlinear mixed-effects model [Equation (16)]. H, tree height; DBH, stem diameter at breast height.

**Figure 5:**
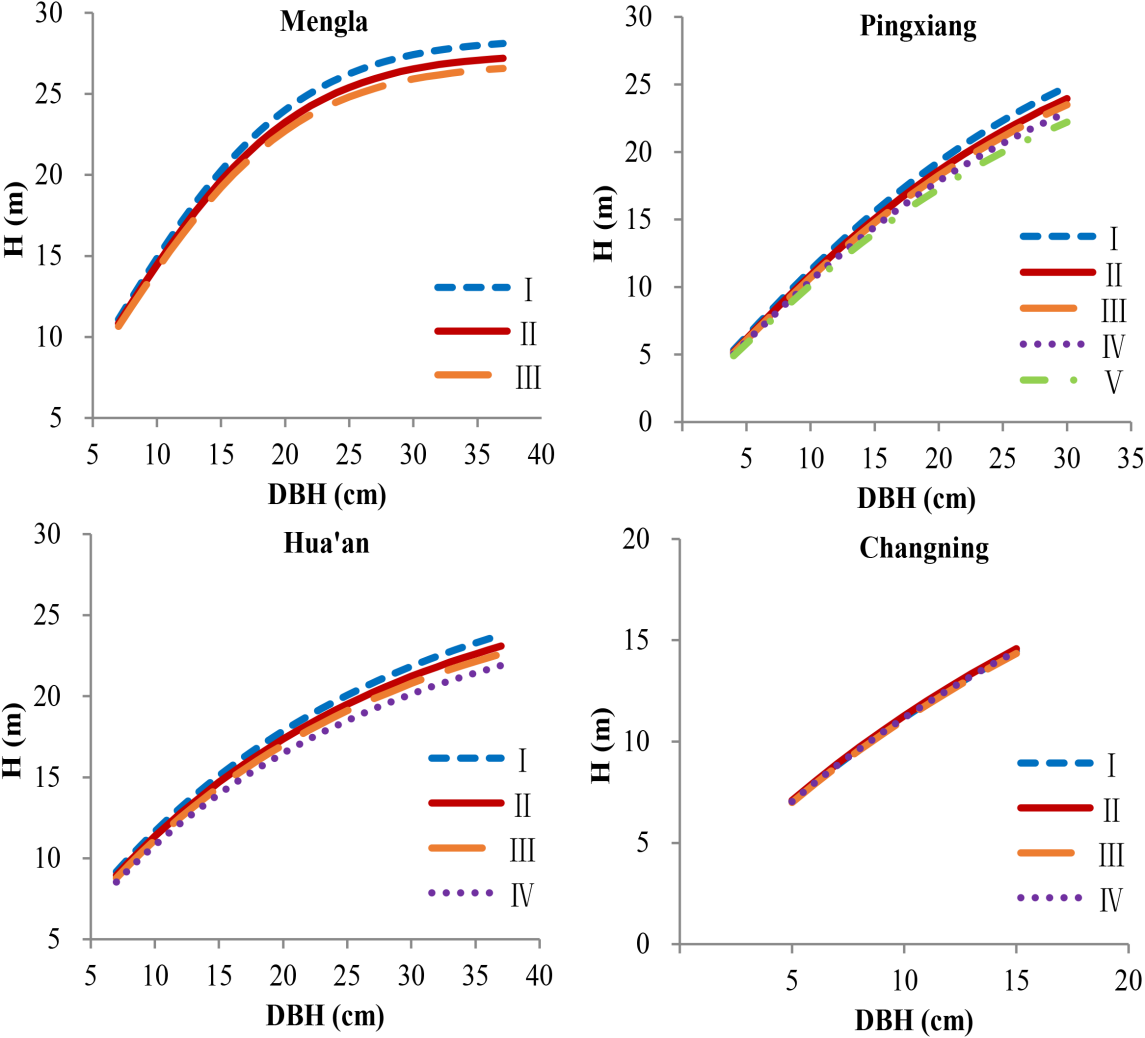
Simulation plots of height–diameter curves for *Betula alnoides* at four sites based on provenance-level mixed-effects model [Equation (17)]. I–V, provenance groups, see **Table 6**; H, tree height.

All the basic estimated parameters ( $\varphi_{0}$ , $\varphi_{1}$ , and $\varphi_{2}$ ) of the models were significantly different from zero (*P<* 0.001), and the parameters $x_{1}$ and $x_{2}$ were also significant (*P<* 0.001 and *P<* 0.05) rather than $x_{3}$ (**Table 4**), indicating that the trees at Mengla and Pingxiang sites were significantly taller than those at Hua’an and Changning sites for a given DBH. The H-D curves at four sites by Equation (16) are shown in **Figure 4**. The simulation results of provenance-level NLME model [Equation (17)] indicated that the influences of provenance variables on asymptote of the models differed among four sites, and difference of asymptote parameters ( $k_{j}$ ) among provenances was more significant at Mengla than other three sites. To demonstrate the variance of the H-D relationships clearly, the provenances were clustered into three to five groups based on asymptote parameters (*k_j_*) of Equation (17) at each site (**Supplementary Figure 1**; **Table 6**). The simulation plots of the H-D curves for these groups were shown in **Figure 5**. The H increased rapidly with increasing DBH at Changning site, whereas it gradually increased slowly at the other sites, and it tended to be stable at Mengla site. The differences of asymptote among provenances increased with increasing DBH and were not obvious at Changning site.

**Table 6:** Growth performances and selected gains (values in brackets are standard error) of the excellent *Betula alnoides* provenances seleted by asymptote parameter (*k_j_*) and stem diameter at breast height (DBH) at four sites.

Sites	Groups	Provenances	Height (m)	DBH (cm)	H-D ratio	Volume (m^3^)	Asymptote parameter *k_j_*
Mengla	I	I, D, A, F, M, C, G, L	19.84 (0.62)	21.20 (1.15)	0.97 (0.02)	0.363 (0.05)	0.944 (0.183)
	II	X, H, K, Y, U, N, J, R	18.78 (0.49)	20.33 (0.90)	0.95 (0.02)	0.312 (0.035)	0.025 (0.138)
	III	P, V, B, S, E, Q, W, T, O	18.38 (0.29)	20.39 (0.60)	0.92 (0.02)	0.305 (0.025)	−0.618 (0.22)
	Total mean		19.00 (0.78)	20.60 (0.95)	0.95 (0.03)	0.324 (0.045)	0.087 (0.681)
	Selected mean	B, W, P, J, N	18.74 (0.16)	20.90 (0.36)	0.92 (0.01)	0.331 (0.014)	−0.338 (0.286)
	Gains (%)		−1.38	1.46	−3.07	1.99	−486.05
Pingxiang	I	A, J, G, M	12.55 (0.60)	12.58 (0.75)	1.01 (0.02)	0.083 (0.016)	1.642 (0.549)
	II	D, V, N, I, T	13.60 (1.48)	14.44 (2.10)	0.96 (0.03)	0.124 (0.050)	0.423 (0.093)
	III	Y, C, B, X, K, H, W	13.47 (1.43)	14.83 (1.91)	0.93 (0.03)	0.133 (0.043)	−0.258 (0.179)
	IV	E, S, P, L, F	13.20 (1.81)	14.84 (2.54)	0.91 (0.04)	0.130 (0.056)	−0.995 (0.236)
	V	Q, O, R, U	13.98 (0.53)	16.73 (1.12)	0.86 (0.03)	0.160 (0.030)	−2.018 (0.112)
	Total mean		13.90 (1.30)	15.60 (2.10)	0.91 (0.05)	0.147 (0.046)	−0.247 (1.177)
	Selected mean	R, P, U, E, W	14.62 (0.27)	17.14 (0.50)	0.87 (0.02)	0.181 (0.011)	−1.286 (0.768)
	Gains (%)		5.18	9.87	−4.72	22.77	−420.67
Hua’an	I	G, P, L, O, H, D, B	17.03 (1.19)	20.41 (2.04)	0.86 (0.03)	0.284 (0.076)	1.548 (0.536)
	II	C, A, M, K	16.46 (0.30)	20.00 (0.75)	0.84 (0.02)	0.258 (0.022)	0.690 (0.092)
	III	I, R, J, U, Y	17.18 (1.07)	22.01 (1.09)	0.80 (0.02)	0.329 (0.047)	0.609 (1.138)
	IV	E, X, T, Q, F, N, W, S, V	15.93 (0.61)	21.09 (1.07)	0.78 (0.03)	0.283 (0.032)	−0.813 (0.581)
	Total mean		16.50 (0.85)	21.10 (1.38)	0.80 (0.04)	0.293 (0.047)	0.270 (1.068)
	Selected mean	Q, R, U, W, J	16.85 (0.34)	22.35 (0.60)	0.77 (0.02)	0.327 (0.020)	−0.269 (0.463)
	Gains (%)		2.11	5.95	−3.99	11.64	−199.46
Changning	I	Q, K, A, I, J	8.64 (0.31)	7.76 (0.41)	1.13 (0.02)	0.021 (0.003)	0.804 (0.106)
	II	S, D, H	8.34 (0.29)	7.67 (0.29)	1.10 (0.00)	0.019 (0.002)	0.467 (0.008)
	III	M, U, L, R, N, B, T, C, X, V	8.34 (0.30)	7.89 (0.42)	1.07 (0.02)	0.021 (0.003)	0.289 (0.064)
	IV	E, Y, P, O, F, W	8.10 (0.11)	7.80 (0.14)	1.06 (0.01)	0.019 (0.001)	−0.022 (0.059)
	Total mean		8.30 (0.31)	7.70 (0.34)	1.11 (0.03)	0.018 (0.003)	0.341 (0.297)
	Selected mean	B, V, T, W, P	8.60 (0.31)	8.21 (0.31)	1.07 (0.03)	0.023 (0.002)	0.316 (0.374)
	Gains (%)		3.59	6.58	−3.81	29.81	−7.17

A, Mengla; B, Yuanyang; C, Mojiang; D, Jinghong; E, Xichou; F, Zhenyuan; G, Tengchong; H, Jinggu; I, Ruili; J, Fengqing; K, Pingbian; L, Jiangcheng; M, Shuangjiang; N, Lancang; O, Lingyun; P, Longzhou; Q, Donglan; R, Tianlin; S, Debao; T, Tiane; U, Pingguo; V, Baise; W, Tianyang; X, Jingxi; and Y, Napo; H-D ratio, tree height to DBH ratio. Excellent provenances are selected at four site based on Model (17).

### 3.4 Excellent provenances selection

The results of elite provenances selection showed that provenances B, W, P, J, and N performed well at Mengla; R, P, U, E, and W did well at Pingxiang; Q, R, U, W, and G did well at Hua’an; and B, V, T, W, and P did well at Changning (**Table 6**). Their asymptotes parameters values ( $k_{j}$ ) and H-D ratio decreased from 7.17% to 486.05% and from 3.07% to 4.72% at four sites, respectively, and the selection gains of H, DBH, and individual volume ranged from −1.38% to 5.18%, from 1.46% to 9.87%, and from 1.99% to 29.81%, respectively.

## 4 Discussion

### 4.1 Model simulation

The application of sigmoidal nonlinear models with inflexion points and asymptotes in simulation height–diameter (H-D) relationship has a long history in tropical and subtropical zones, and many nonlinear models have been developed (Scaranello et al., 2012). The study of Scaranello et al. (2012) indicated that Weibull and Chapman–Richards nonlinear models could improve the fitting accuracy of the H-D relationship in biomass estimates for tropical Atlantic forests in southeastern Brazil. Linear models are also suitable tools once a linearizing relationship can be established through data transformation and reasonable biological significance can be interpreted (Curtis, 1967; Watt and Kirschbaum, 2011). One typical example was that Buford (1986) used transformed linear models to fit the H-D relationship of loblolly pine and found out that the R^2^ values were consistently larger than 0.88, which provided an appropriate and accurate simulation of height–diameter curves. In the present study, Weibull nonlinear model [Equation (8)] showed the best goodness of fit (R^2 = ^0.837) among 10 candidate models when fitted with the dataset of H and DBH and was selected as a base H-D model for *B. alnoides*.

Whether based on linear or nonlinear models, the way to further improve the goodness of fit is to introduce and explain more effect variables (Zhang et al., 2019). The mixed model and dummy variable model approaches are frequently used to construct the H-D relationship equations for tree species. Mixed-effects models can isolate fixed and random variations and are applied to incorporate complex nested stochastic structure in the H-D curves. For instance, Zhang et al. (2019) introduced climate, site, plot variables, etc., into the NLME model to reveal the influence factors on the H-D relationship of Chinese fir. Dummy variable models are also of adequate methodology, especially for the quantification of categorical variables. For an example, Buford (1986) introduced locations into the linear model as dummy variables and revealed clearly the variation of asymptotes in H-D curves among locations.

For the variation analysis of multi-layer complex variables, both dummy variables and mixed-effects models are all powerful modeling tools. There is a little difference between the two kinds of models when the number of samples in each category is large (Wang et al., 2008; Fu et al., 2012). In the present study, the dataset contained 953–3,403 individuals at four site with average of 38–136 individuals per provenance at each site (**Table 2**), and both dummy variables model and mixed-effects model were thus applied comprehensively. Sites and provenances were introduced into the base model as dummy variables, and block was introduced as random effect to develop the nonlinear mixed-effects models. Significant variations of the H-D relationship were then observed among sites and provenances at each site, and the clear visualization of variation for the H-D relationship among provenances would provide a great convenience for the following selection breeding for *B. alnoides*.

### 4.2 Site effect

A better performance of height growth and higher asymptote of the H-D curves was observed, and the H was infinitely close to the growth limit at Mengla, indicating that better soil nutrients and water conditions might be conducive to realise the genetic potential of H in the present study. A study on Norway spruce indicated that abundant soil water and nutrients were beneficial to height growth and thus had a high asymptote of the H-D curves (Bošeľa et al., 2014). In the study of Trouvé et al. (2015) on *Quercus petraea* Liebl. and the study of Kempes et al. (2011) on the forests in the continental United States, H growth usually decreased and more resources were assigned into radial growth with increasing water stress, which could also result in a low asymptote of the H-D curves. Zhang et al. (2019) reported that the H of Chinese fir plantations decreased with increasing annual heat moisture index in the subtropical zone. These also verify that sufficient water and nutrients can promote H growth and affect the H-D curves. The study of Homeier et al. (2010) on Ecuadorian montane rain forest and the study of Bošeľa et al. (2014) on Norway spruce in the Western Carpathians demonstrated that H tended to decrease with increasing altitude, and DBH gradually played a more important role than H in the H-D relationship. In the present study, the H-D relationship did not show a clear trend with such a large range variation of altitude (275–1250 m) at four sites. This might be closely dependent on the complex geomorphic features in southern China. Buford (1986) found that the asymptotes of the H-D curves for loblolly pine were different across six sites based on a dummy variable model. The site variables used in the present study are also a general one, and specific variables of site conditions should be included to improve the model accuracy and reveal influencing factors in further studies.

### 4.3 Provenance effect

The variation of the H-D relationship among provenances was not consistent at four sites, demonstrating that there were interactions between provenances and sites. This genotype by environment interaction of other growth and quality traits was also observed in our previous study (Yin et al., 2019). The differences among provenances were modeled separately for each site to deal with this interaction in the present study. The specific expression of genetic effect was that different asymptotes and similar slopes of the H-D curves were seen among provenances at each sites. This was following the study of Buford (1986) on loblolly pine at the age of 15 years, in which six of the nine sites showed a provenance effect on the asymptotes of the H-D curves, whereas the study of Egbäck et al. (2015) on loblolly pine at the age of 6 years indicated that there were no significant differences among one seed orchard mix and six families for both the asymptote and slope parameters from the Korf function. This inconsistency of genetic variance may be due to the facts that the differences are larger at provenance level than those at family level, and more genetic differences of the H-D relationship are expressed at the age of 15 years than those at the age of 6 years. In the present study, the variance of asymptotes of the H-D relationship among provenances was relatively smaller in Changning site at the age of 10 years compared with that in other sites at the age of 14–15.

### 4.4 Superior provenance selection

High selective gain is the core purpose of the selective breeding process. In the present study, five excellent provenances were selected on the basis of the lower asymptotes [*k_j_* in Equation (17)] of the H-D curves and DBH above mean values at each site. The selection gains of DBH, height, and individual volume ranged from 1.46% to 9.87%, from −1.38% to 5.18%, and from 1.99% to 29.81%, respectively, for four sites in the present study, whereas they were 7.31%, 4.57%, and 14.84% based on growth and quality traits in our previous study (Yin et al., 2019). Although DBH, height, and individual volume varied among the sites, the maximum selection gains of them in the present study were all higher than those in the previous study. The selection gains of DBH, height, and individual volume at Mengla were lower than those at the other three sites. This could be attributed to the trade-off between lower asymptote and biger DBH of provenances at Mengla site, in which the fertile soil and suitable climate formed biger DBH and also higher tree hight and asymptote of provenances.

Although there is a statistical correlation between DBH and H, the disproportional selection of DBH and H will still occur in the selection process. For example, Yin et al. (2019), using biplot analysis for main genotypic effects and G × E interaction to select the superior provenances of *B. alnoides*, found that the selection gains of DBH (7.31%) were much higher than that of H (4.57%), whereas Wang et al. (2017) observed that the superior clones’ selection gain of H (14.84%) was much higher than that of DBH (3.95%) during selecting superior clones of *B. alnoides* based on simple index method. When selecting through volume alone in the present study, the selection gain of DBH (6.0%–10.8%) was higher than that of H (5.3%–8.1%) (**Supplementary Table 2**). These inferred that excellent germplasms with high selection gains in the H-D relationship could not be obtained through disproportional selection of DBH and H.

The selection results of DBH and H can affect the H-D ratio, which is related to the stem quality and stand stability (Coutts, 1986; Sharma et al., 2016; Zhang et al., 2020). Schmidt and Kändler (2009) once found that the individuals of the higher site index had the lower H-D ratios for Norway spruce; and for a given site index, the lower the H-D ratios, the higher the stem quality. Tree pulling experiments conducted by Peltola et al. (2000) also showed that individuals of birch species (*Betula* spp.) with the lower H-D ratios had the bigger resistive bending moment for stem breakage. Therefore, excellent germplasms with lower H-D ratios have more production value in plantation management. Moreover, H-D ratio is a factor which should be taken into account during selecting excellent germplasms. Because of the disproportional selection of DBH and H, the excellent germplasms usually had a higher H-D ratio than the unselected germplasm in previous studies. Andersson et al. (2007) and Kroon et al. (2008) all observed that the selection for improved growth, based on H, resulted in trees with a higher H-D ratio. Similarly, Sabatia and Burkhart (2013) also found that the average H of the improved loblolly pine clones increased, whereas the average DBH did not, because the H-D relationship was not considered. Therefore, they also pointed out the high necessity of introducing the H-D relationship into the selection methods system of superior germplasm.

In the present study, the H-D ratios of the superior germplasms reduced from 3.07% to 4.72% at four sites based on selection by asymptote parameter (*k_j_*) and DBH. While after calculating the selection gains of the H-D ratios in the excellent germplasms of our previous study (Yin et al.2019), we found they were −1.7%, −0.8%, 2.5%, and −0.5% in Mengla, Pingxiang, Hua’an, and Changning, respectively (not published). When using volume for selection alone in the present study (**Supplementary Table 2**), the modified direction of the H-D ratios was also unstable, which increased at Mengla site with the percentages of 0.84% but reduced from 0.39% to 3.00% at other three sites due to improvement prior to DBH. Their asymptote parameter (*k_j_*) was all increased significantly from 37.73% to 1023.22% at four sites. The inconsistency between H-D ratio and asymptote parameter (*k_j_*) was attributable to the fact that the H-D ratio is not constant over time and may include scaling effects (Kroon et al., 2008). These also proved the superiority of asymptote parameter (*k_j_*) in selecting superior germplasms by the H-D relationship. As a whole, selecting excellent provenances with the lower asymptote parameter (*k_j_*) of the H-D curves and above-average DBH was a logical and valid genetic improvement strategy. In addition, further studies should be carried out on linking stem form factor with the H-D relationship to predict volume gains more accurately.

## 5 Conclusions

The H-D relationship was modeled for 25 provenances of *B. alnoides* at four sites at the ages of 10–15 years in the present study. On our opinion, it is the fist report on genetic improvement with the H-D relationship involved for hardwood species. Among 10 candidate models, Weibull model was selected as the base model for the H-D relationship because of its best goodness of fit. The further dummy variable NLME models showed that there exist significant site, provenance, and provenance-site effects for the H-D relationship. The asymptotes of the H-D curves were affected by site, and the provenance effect on the H-D relationship varied across sites. Five superior provenances were selected on the basis of asymptote of the H-D curves and DBH at each site, which can be applied in large-sized timber production of *B. alnoides*. Their selection gains of individual volume ranged from 1.99% to 29.81%, and those of asymptote parameter (*k_j_*) and H-D ratio decreased from 7.17% to 486.05% and from 3.07% to 4.72% at four sites, respectively.

## Data availability statement

The raw data supporting the conclusions of this article will be made available by the authors, without undue reservation.

## Author contributions

MY: investigation, data curation, formal analysis, visualization, and writing—original draft; JG: conceptualization, project administration, investigation, methodology, and writing—review and editing; CW: methodology, formal analysis, and visualization; HW and ZZ: methodology and visualization; HQ: formal analysis and visualization; CT: methodology and formal analysis; JZ: conceptualization, resources, methodology, and writing—review and editing. All authors contributed to the article and approved the submitted version.

## References

[B1] Andersson B. Elfving B. Persson T. Ericsson T. Kroon J. (2007). Characteristics and development of improved *Pinus sylvestris* in northern Sweden. Can. J. For. Res. 37, 84–92. doi: 10.1139/x06-224

[B2] Bi H. Q. Fox J. C. Li Y. Lei Y. C. Pang Y. (2012). Evaluation of nonlinear equations for predicting diameter from tree height. Can. J. For. Res. 42, 789–806. doi: 10.1139/x2012-019

[B3] Bošeľa M. Konôpka B. Šebeň V. Vladovič J. Tobin B. (2014). Modelling height to diameter ratio-an opportunity to increase Norway spruce stand stability in the Western Carpathians. Lesn. Cas. For. J. 60, 71–80. doi: 10.2478/forj-2014-0007

[B4] Bošeľa M. Máliš F. Kulla L. Šebeň V. Deckmyn G. (2013). Ecologically based height growth model and derived raster maps of Norway spruce site index in the Western Carpathians. Eur. J. For. Res. 132, 691–705. doi: 10.1007/s10342-013-0708-z

[B5] Buford M. A. (1986). Height-diameter relationships at age 15 in 1oblolly pine seed sources. For. Sci. 32, 812–818.

[B6] Buford M. A. Burkhart H. E. (1987). Genetic improvement effects on growth and yield of loblolly pine plantations. For. Sci. 33, 707–724.

[B7] Carson S. D. Garcia O. Hayes J. D. (1999). Realized gain and prediction of yield with genetically improved *Pinus radiata* in New Zealand. For. Sci. 45, 186–200. doi: 10.1093/forestscience/45.2.186

[B8] Coutts M. P. (1986). Components of tree stability in Sitka Spruce on peaty gley soil. Forestry 59, 173–197. doi: 10.1093/forestry/59.2.173

[B9] Curtis R. O. (1967). Height-diameter and height-diameter-age equations for second-growth Douglas-fir. For. Sci. 13, 365–375. doi: 10.1515/hfsg.1968.22.6.190

[B10] Davisian M. Giltinan D. M. (1995). Nonlinear models for repeated measurement data (London, UK: Chapman and Hall), 360.

[B11] Ducey M. J. (2012). Evergreenness and wood density predict height–diameter scaling in trees of the northeastern United States. For. Ecol. Manage. 279, 21–26. doi: 10.1016/j.foreco.2012.04.034

[B12] Egbäck S. Bronson P. B. Isik F. Mckeand S. E. (2015). Height-diameter relationships for different genetic planting stock of loblolly pine at age 6. For. Sci. 61 (3), 424–425. doi: 10.5849/forsci.14-015

[B13] Egbäck S. Karlsson B. Högberg K.-A. Nyström K. Nilsson U. (2018). Effects of phenotypic selection on height-diameter ratio of Norway spruce and Scots pine in Sweden. Silva. Fenn. 52, 1–15. doi: 10.14214/sf.7738

[B14] Fang Z. Bailey R. L. (2001). Nonlinear mixed-effect modeling for slash pine dominant height growth following intensive silvicultural treatments. For. Sci. 47, 287–300. doi: 10.1046/j.1439-0329.2001.00240.x

[B15] Feldpausch T. R. Banin L. Phillips O. L. Baker T. R. Lewis S. L. Quesada C. A.. (2011). Height-diameter allometry of tropical forest trees. Biogeosciences 8, 1081–1106. doi: 10.5194/bg-8-1081-2011

[B16] Fu L. Y. Sun W. Wang G. X. (2016). A climate-sensitive aboveground biomass model for three larch species in northeastern and northern China. Trees 31, 557–573. doi: 10.1007/s00468-016-1490-6

[B17] Fu L. Y. Zeng W. S. Tang S. Z. Sharma R. P. Li H. K. (2012). Using linear mixed model and dummy variable model approaches to construct compatible single-tree biomass equations at different scales – A case study for Masson pine in Southern China. J. For. Sci. 58, 101–115. doi: 10.17221/69/2011-JFS

[B18] Gould P. J. Marshall D. D. (2010). Incorporation of genetic gain into growth projections of Douglas-fir using ORGANON and the forest vegetation simulator. West. J. Appl. For. 25, 55–61. doi: 10.1093/wjaf/25.2.55

[B19] Guo W. F. Zeng J. Li M. (2008). Provenance and family trials for Betula alnoides in Pingxiang Guangxi province I. early variation of growth traits. For. Res. 21, 652–656.

[B20] Henriksen H. A. (1950). Height/diameter curve with logarithmic diameter: brief report on a more reliable method of height determination from height curves, introduced by the State Forest Research Branch. Dansk Skovforeningens Tidsskrift 35, 193–202.

[B21] Homeier J. Breckle S. W. Günter S. Rollenbeck R. T. Leuschner C. (2010). Tree diversity, forest structure and productivity along altitudinal and topographical gradients in a species-rich Ecuadorian montane rain forest. Biotropica 42, 140–148. doi: 10.1111/j.1744-7429.2009.00547.x

[B22] Huang S. Titus S. J. (1992). Comparison of nonlinear height-diameter functions for major Alberta tree species. Can. J.For. Res. 22, 1297–1304. doi: 10.1139/x92-172

[B23] Hulshof C. M. Swenson N. G. Weiser M. D. (2015). Tree height-diameter allometry across the United States. Ecol. Evol. 5, 1193–1204. doi: 10.1002/ece3.1328 25859325 PMC4377263

[B24] Kempes C. P. West G. B. Crowell K. Girvan M. (2011). Predicting maximum tree heights and other traits from allometric scaling and resource limitations. PloS One 6, 1–10. doi: 10.1371/journal.pone.0020551 PMC3113805 21695189

[B25] Kroon J. Andersson B. Mullin T. J. (2008). Genetic variation in the diameter–height relationship in Scots pine (*Pinus sylvestris*). Can. J. For. Res. 38, 1493–1503. doi: 10.1139/X07-233

[B26] Lei X. D. Peng C. H. Wang H. Y. Zhou X. L. (2009). Individual height-diameter models for young black spruce (*Picea mariana*) and jack pine (*Pinus banksiana*) plantations in New Brunswick. Canada. Forest. Chron. 85, 43–56. doi: 10.5558/tfc85043-1

[B27] Li X. F. Tang S. Z. Yuan G. R. Ban D. C. (1994). Self-adjusted height-diameter curves and one-entry volume model. For. Res. 7, 512–518.

[B28] Liu M. Feng Z. K. Zhang Z. X. Ma C. H. Zhang L. (2017). Development and evaluation of height diameter at breast models for native Chinese Metasequoia. PloS One 12, e0182170. doi: 10.1371/journal.pone.0182170 28817600 PMC5560716

[B29] Pearl R. Reed L. J. (1920). On the rate of growth of the population of the United States since 1790 and its mathematical representation. Proc. Natl. Acad. Sci. U.S.A. 6, 275–288. doi: 10.1073/pnas.6.6.275 16576496 PMC1084522

[B30] Peltola H. Kellomäki S. Hassinen A. Granander M. (2000). Mechanical stability of Scots pine, Norway spruce and birch: an analysis of tree-pulling experiments in Finland. For. Ecol. Manage. 135, 143–153. doi: 10.1016/S0378-1127(00)00306-6

[B31] Pinheiro J. Bates D. DebRoy S. Sarkar D. R Core Team (2017) nlme: Linear and nonlinear mixed effects models. R package persion. Available at: https://CRAN.R-project.org/package=nlme (Accessed 6 February, 2017).

[B32] Price C. A. Enquist B. J. Savage M. (2007). A general model for allometric covariation in botanical form and function. Proc. Natl. Acad. Sci. U.S.A. 104, 13204–13209. doi: 10.1073/pnas.0702242104 17664421 PMC1941814

[B33] Raj A. D. A. Malarvili T. Velavan S. (2015). Restorative effect of *Betula alnoides* bark on hepatic metabolism in high fat diet fed Wistar rats. Int. J. Pharma Biosci. 6, 1281–1288.

[B34] Richards F. J. (1959). A flexible growth function for empirical use. J. Exp. Bot. 10, 290–301. doi: 10.1093/jxb/10.2.290

[B35] Richter C. (2015). “Wood characteristics inherent in a tree's natural growth,” in Wood Characteristics (Cham, Switzerland: Springer), 35–124.

[B36] Robichaud E. Methven I. R. (1991). Tree vigor and height growth in Black Spruce. Trees 5, 158–163. doi: 10.1007/BF00204338

[B37] Sabatia C. O. Burkhart H. E. (2013). Height and diameter relationships and distributions in loblolly pine stands of enhanced genetic material. For. Sci. 59, 278–289. doi: 10.5849/forsci.11-093

[B38] Scaranello M. A. D. S. Alves L. F. Vieira S. A. Camargo P. B. D. Joly C. A. Martinelli L. A. (2012). Height-diameter relationships of tropical Atlantic moist forest trees in southeastern. Brazil. Sci. Agric. 69, 26–37. doi: 10.1590/S0103-90162012000100005

[B39] Schmidt M. Kändler G. (2009). An analysis of Norway spruce stem quality in Baden-Württemberg: results from the second German national forest inventory. Eur. J. For. Res. 128, 515–529. doi: 10.1007/s10342-009-0301-7

[B40] Sharma R. P. Vacek Z. Vacek S. (2016). Modeling individual tree height to diameter ratio for Norway spruce and European beech in Czech Republic. Trees 30, 1969–1982. doi: 10.1007/s00468-016-1425-2

[B41] Stage A. R. (1975). Prediction of height increment for models of forest growth. USDA For. Ser. Res. Paper INT-164. doi: 10.5962/bhl.title.69034

[B42] Sur T. K. Pandit S. Battacharyya D. Kumar C. K. A. Lakshmi S. M. Chatttopadhyay D. (2002). Studies on the anti-inflammatory activity of *Betula alnoides* bark. Phytother. Res. 16, 669–671. doi: 10.1002/ptr.942 12410550

[B43] Temesgen H. Zhang C. H. Zhao X. H. (2014). Modelling tree height-diameter relationships in multi-species and multi-layered forests: A large observational study from Northeast China. For. Ecol. Manage. 316, 78–89. doi: 10.1016/j.foreco.2013.07.035

[B44] Trouvé R. Bontemps J. D. Seynave I. Catherine C. Lebourgeois F. (2015). Stand density, tree social status and water stress influence allocation in height and diameter growth of *Quercus petraea* (Liebl.). Tree Physiol. 35, 1035–1046. doi: 10.1093/treephys/tpv067 26232785

[B45] Valinger E. Fridman J. (2011). Factors affecting the probability of wind throw at stand level as a result of Gudrun winter storm in southern Sweden. For. Ecol. Manage. 262, 398–403. doi: 10.1016/j.foreco.2011.04.004

[B46] Vanclay J. K. (1995). Growth models for tropical forests: A synthesis of models and methods. For. Sci. 41, 7–42. doi: 10.1093/forestscience/41.1.7

[B47] Vergara R. White T. L. Huber D. A. Shiver B. D. Rockwood D. L. (2004). Estimated realized gains for first-generation slash pine (*Pinus elliottii* var. *elliottii*) tree improvement in the southeastern United States. Can. J. For. Res. 34, 2587–2600. doi: 10.1139/x04-136

[B48] Vizcaíno-Palomar N. Ibáñez I. Benito-Garzón M. González-Martínez S. C. Zavala M. A. Alía R. (2016). Climate and population origin shape pine tree height-diameter allometry. New Forest. 48, 363–379. doi: 10.1007/s11056-016-9562-4

[B49] Wang M. Borders B. E. Zhao D. (2008). An empirical comparison of two subject-specific approaches to dominant heights modeling the dummy variable method and the mixed model method. For. Ecol. Manage. 255, 2659–2669. doi: 10.1016/j.foreco.2008.01.030

[B50] Wang X. Fang J. Tang Z. Zhu B. (2006). Climatic control of primary forest structure and DBH-height allometry in Northeast China. For. Ecol. Manage. 234, 264–274. doi: 10.1016/j.foreco.2006.07.007

[B51] Wang C. S. Hein S. Zhao Z. G. Guo J. J. Zeng J. (2016). Branch occlusion and discoloration of *Betula alnoides*, under artificial and natural pruning. For. Ecol. Manage. 375, 200–210. doi: 10.1016/j.foreco.2016.05.027

[B52] Wang H. Zeng J. X. Luo B. G. Guo J. J. Wang C. S. Zhao Z. G. (2017). Multiple-trait combined selection of superior *Betula alnoides* clones in eastern Guangdong. J. Cent. South Univ. For. Tech. 37, 72–75, 84. doi: 10.14067/j.cnki.1673-923x.2017.12.012

[B53] Wang C. S. Zhao Z. G. Zeng J. Guo J. J. Sha E. Guo W. F. (2013). Relationship between planting density and tree growth process of *Betula alnoides* mid-young plantation in Pingxiang’ Guangxi. For. Res. 26, 257–262. doi: 10.13275/j.cnki.lykxyj.2013.02.020

[B54] Watt M. S. Kirschbaum M. U. F. (2011). Moving beyond simple linear allometric relationships between tree height and diameter. Ecol. Model. 222, 3910–3916. doi: 10.1016/j.ecolmodel.2011.10.011

[B55] Weng Y. H. Kershaw J. Tosh K. Adams G. Fullarton M. S. (2008). Height-diameter delationships for Jack pine seedlots of different genetic improvement levels. Silvae Genet. 57, 276–282. doi: 10.1515/sg-2008-0042

[B56] Wykoff W. R. Crookston N. L. Stage A. R. (1982). User's guide to the stand prognosis model. USDA For. Ser. Gen. Tech. Rep. INT-133. doi: 10.2737/INT-GTR-133

[B57] Yang Y. P. Guo J. J. Huang J. C. Zhao Z. G. Zhou Z. M. Zeng J. (2012). Early choose of *Betula alnoides* provenance and family in western part of Yunnan province. Seed 31, 67–70. doi: 10.16590/j.cnki.1001-4705.2012.01.074

[B58] Yang R. C. Kozak A. Smith J. H. G. (1978). The potential of weibull-type functions as a flexible growth curves. Can. J. For. Res. 8, 424–431. doi: 10.1139/x78-062

[B59] Yih L. T. Kershaw J. A. Nur H. Z. S. Abd R. K. Weiskittel A. R. Potts M. D. (2017). Evaluating and modelling genus and species variation in height-to-diameter relationships for Tropical Hill Forests in Peninsular Malaysia. Forestry 90, 268–278. doi: 10.1093/forestry/cpw051

[B60] Yin M. Y. Guo J. J. Wang C. S. Zhao Z. G. Zeng J. (2019). Genetic Parameter Estimates and Genotype×Environment Interactions of Growth and Quality Traits for *Betula alnoides* Buch-Ham. ex D. Don in Four Provenance-Family Trials in Southern China. Forests 10, 1036. doi: 10.3390/f10111036

[B61] Zeng J. Wang Z. R. Zhou S. L. Bai J. Y. Zheng H. S. (2003). Allozyme variation and population genetic structure of *Betula alnoides* from Guangxi, China. Biochem. Genet. 41, 61–75. doi: 10.1023/A:1022027832065 12670021

[B62] Zeng J. Zheng H. S. Weng Q. J. (1999). Geographic distributions and ecological conditions of *Betula alnoides* in China. For. Res. 12, 479–484.

[B63] Zhang X. Q. Chhin S. Fu L. Y. Lu L. Duan A. G. Zhang J. G. (2019). Climate-sensitive tree height–diameter allometry for Chinese fir in southern China. Forestry 92, 167–176. doi: 10.1093/forestry/cpy043

[B64] Zhang X. Q. Duan A. G. Zhang J. G. Xiang C. W. (2014). Estimating tree height-diameter models with the Bayesian method. Sci. World J. 2014, 683691. doi: 10.1155/2014/683691 PMC3953559 24711733

[B65] Zhang X. Q. Wang H. C. Chhin S. Zhang J. G. (2020). Effects of competition, age and climate on tree slenderness of Chinese fir plantations in southern China. For. Ecol. Manage. 458, 117815. doi: 10.1016/j.foreco.2019.117815

