# Supplementary material for: Modeling and evaluating site and provenance variation in height–diameter relationships for Betula alnoides Buch.–Ham. ex D. Don in southern China
Source: Front Plant Sci. 2023 Oct 2;14:1248278. doi: 10.3389/fpls.2023.1248278 (PMC10577385; doi:10.3389/fpls.2023.1248278)
Supplement: Supplementary file 1 [file DataSheet_1.docx]

**Table S1** Voucher specimens information for 25 provenances of *Betula alnoids* in this study.

| Provenances | Location | Voucher specimen accession no. ^a^ | Identifier |
| --- | --- | --- | --- |
| A | Mengla, Yunnan | BA-RITF-YNML-01 | Jie Zeng |
| B | Yuanyang, Yunnan | BA-RITF-YNYY-03 | Jie Zeng |
| C | Mojiang, Yunnan | BA-RITF-YNMJ-01 | Jie Zeng |
| D | Jinghong, Yunnan | BA-RITF-YNJH-01 | Jie Zeng |
| E | Xichou, Yunnan | BA-RITF-YNXC-01 | Jie Zeng |
| F | Zhenyuan, Yunnan | BA-RITF-YNZY-01 | Jie Zeng |
| G | Tengchong, Yunnan | BA-RITF-YNTC-11 | Jie Zeng |
| H | Jingguo, Yunnan | BA-RITF-YNJG-01 | Jie Zeng |
| I | Ruili, Yunnan | BA-RITF-YNRL-02 | Jie Zeng |
| J | Fengqing, Yunnan | BA-RITF-YNFQ-01 | Jie Zeng |
| K | Pingbian, Yunnan | BA-RITF-YNPB-01 | Jie Zeng |
| L | Jiangcheng, Yunnan | BA-RITF-YNJC-02 | Jie Zeng |
| M | Shuangjiang, Yunnan | BA-RITF-YNSJ-04 | Jie Zeng |
| N | Lancang, Yunnan | BA-RITF-YNLC-01 | Jie Zeng |
| O | Lingyun, Gaungxi | BA-RITF-GXLY-01 | Jie Zeng |
| P | Longzhou, Gaungxi | BA-RITF-GXLZ-02 | Jie Zeng |
| Q | Donglan, Gaungxi | BA-RITF-GXDL-01 | Jie Zeng |
| R | Tianlin, Gaungxi | BA-RITF-GXTL-07 | Jie Zeng |
| S | Debao, Gaungxi | BA-RITF-GXDB-01 | Jie Zeng |
| T | Tian’e, Gaungxi | BA-RITF-GXTE-01 | Jie Zeng |
| U | Pingguo, Gaungxi | BA-RITF-GXPG-01 | Jie Zeng |
| V | Baise, Gaungxi | BA-RITF-GXBS-05 | Jie Zeng |
| W | Tianyang, Gaungxi | BA-RITF-GXTY-03 | Jie Zeng |
| X | Jingxi, Gaungxi | BA-RITF-GXJX-01 | Jie Zeng |
| Y | Napo, Gaungxi | BA-RITF-GXNP-02 | Jie Zeng |

Note: ^a^ BA= *Betula alnoids*; RITF = Herbarium of the Research Institute of Tropical Forestry.

**Table S2** Growth performances and selected gains of the excellent *Betula alnoides* provenances seleted by volume at four sites.

| Sites | Parameters | Height (m) | DBH (cm) | H-D ratio | Volume (m^3^) | Asymptote parameter *k_j_* |
| --- | --- | --- | --- | --- | --- | --- |
| Mengla | Total mean | 19.00(0.78) | 20.60(0.95) | 0.95(0.03) | 0.324(0.045) | 0.087(0.681) |
|  | Mean for excellent provenances | 20.19(0.26) | 21.84(0.64) | 0.96(0.02) | 0.390(0.032) | 0.982(0.208) |
|  | Gains (%) | 6.23 | 6.15 | 0.84 | 20.43 | 1023.22 |
| Pingxiang | Total mean | 13.90(1.30) | 15.60(2.10) | 0.91(0.05) | 0.147(0.046) | -0.247(1.177) |
|  | Mean for excellent provenances | 15.02(0.46) | 17.28(0.42) | 0.89(0.03) | 0.189(0.008) | -0.661(0.983) |
|  | Gains (%) | 8.06 | 10.77 | -3.00 | 28.67 | 167.69 |
| Hua’an | Total mean | 16.50(0.85) | 21.10(1.38) | 0.80(0.04) | 0.293(0.047) | 0.270(1.068) |
|  | Mean for excellent provenances | 17.68(0.91) | 22.84(0.51) | 0.79(0.03) | 0.361(0.028) | 0.781(1.337) |
|  | Gains (%) | 7.40 | 8.14 | -1.47 | 23.07 | 188.83 |
| Changning | Total mean | 8.30(0.31) | 7.70(0.34) | 1.11(0.03) | 0.018(0.003) | 0.341(0.297) |
|  | Mean for excellent provenances | 8.74(0.22) | 8.26(0.29) | 1.08(0.03) | 0.024(0.001) | 0.470(0.305) |
|  | Gains (%) | 5.82 | 6.64 | -0.39 | 21.44 | 37.73 |

Notes: DBH, stem diameter at breast height; H-D ratio, tree height to DBH ratio.

**
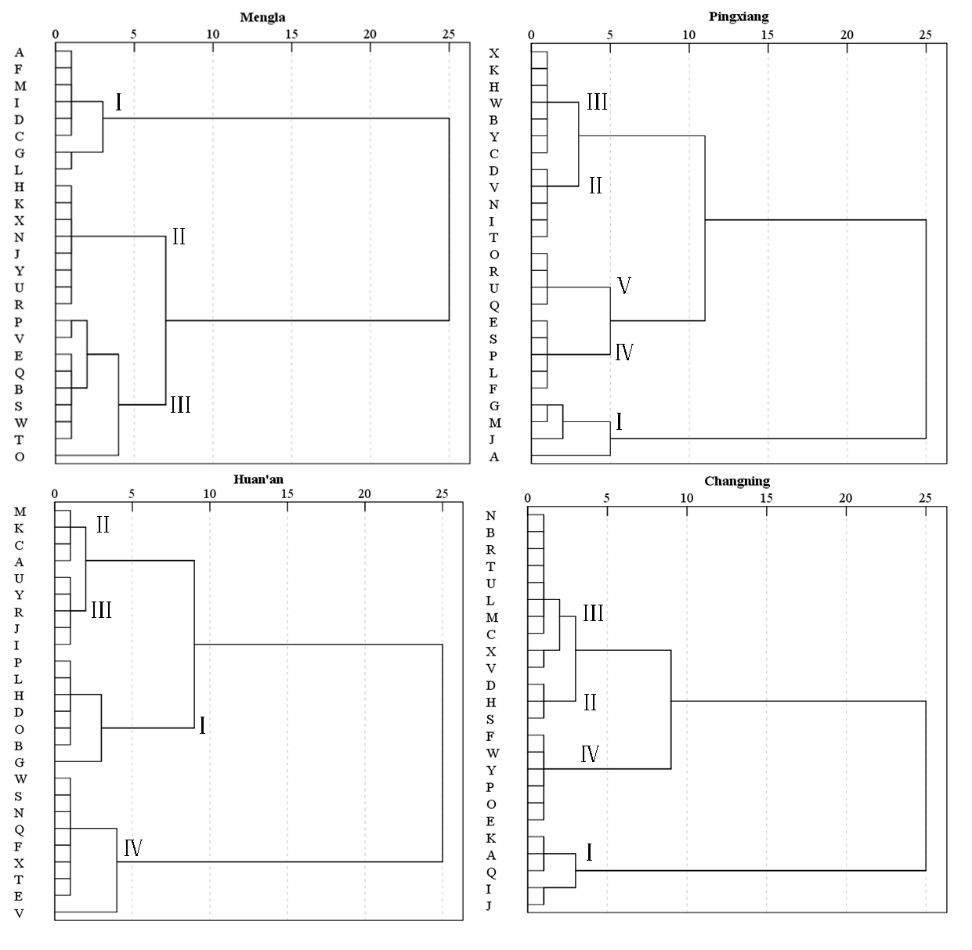
**

**Figure S1** Clustering analysis of 25 provrnances based on Euclidean distance of asymptote parameter ($k_{j}$) by between-groups linkage method at four sites. See Table S1 for provenance codes.
